# Supplementary material for: RNA structure prediction using positive and negative evolutionary information
Source: PLoS Comput Biol. 2020 Oct 30;16(10):e1008387. doi: 10.1371/journal.pcbi.1008387 (PMC7657543; doi:10.1371/journal.pcbi.1008387)
Supplement: S1 File — Includes alignments, and source code. (GZ) [file pcbi.1008387.s008.tar.gz › supplemental_material/src/rscape_v1.5.4.tar.gz!/rscape_v1.5.4/lib/infernal/infernal-1.1.2/documentation/userguide/Figures/parsetrees.pdf]

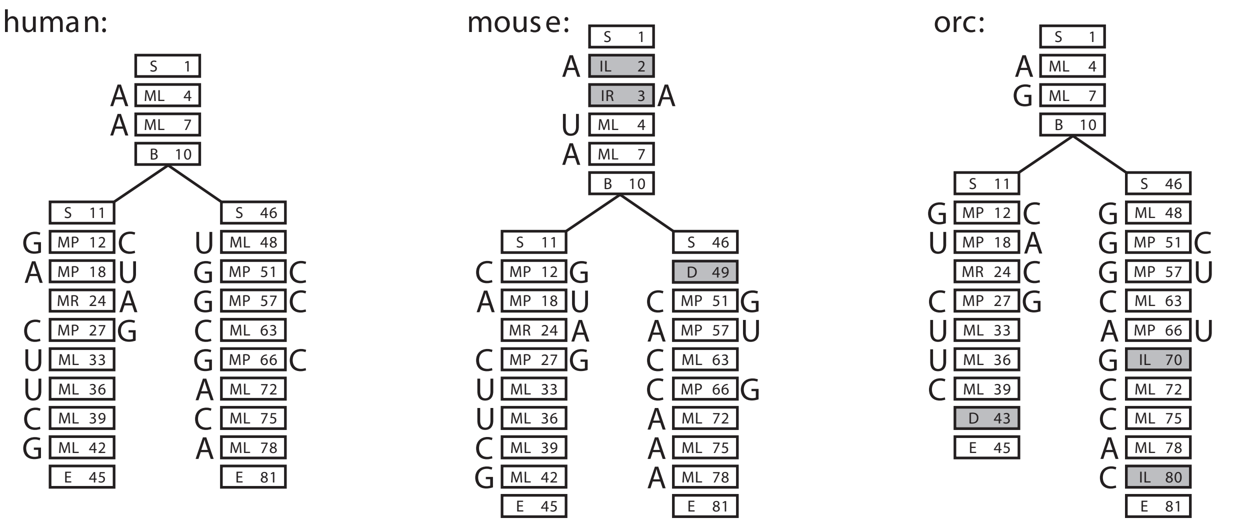

human:
S 1
A ML 4
A ML 7
B 10
S 11
G MP 12 C
A MP 18 U
MR 24 A
C MP 27 G
U ML 33
U ML 36
C ML 39
G ML 42
E 45
S 46
U ML 48
G MP 51 C
G MP 57 C
C ML 63
G MP 66 C
A ML 72
C ML 75
A ML 78
E 81
mouse:
S 1
A IL 2
IR 3 A
U ML 4
A ML 7
B 10
S 11
C MP 12 G
A MP 18 U
MR 24 A
C MP 27 G
U ML 33
U ML 36
C ML 39
G ML 42
E 45
S 46
D 49
C MP 51 G
A MP 57 U
C ML 63
C MP 66 G
A ML 72
A ML 75
A ML 78
E 81
orc:
S 1
A ML 4
G ML 7
B 10
S 11
G MP 12 C
U MP 18 A
MR 24 C
C MP 27 G
U ML 33
U ML 36
C ML 39
D 43
E 45
S 46
G ML 48
G MP 51 C
G MP 57 U
C ML 63
A MP 66 U
G IL 70
C ML 72
C ML 75
A ML 78
C IL 80
E 81
